# Development, Implementation, and Evaluation of an Interdisciplinary Theory- and Evidence-Based Intervention to Prevent Childhood Obesity: Theoretical and Methodological Lessons Learned

**DOI:** 10.3389/fpubh.2017.00352

**Published:** 2017-12-22

**Authors:** Gill A. ten Hoor, Guy Plasqui, Annemie M. W. J. Schols, Gerjo Kok

**Affiliations:** ^1^Department of Human Biology and Movement Sciences, Nutrition and Translational Research in Metabolism, Maastricht University Medical Centre, Maastricht, Netherlands; ^2^Department of Work and Social Psychology, Maastricht University, Maastricht, Netherlands; ^3^Department of Respiratory Medicine, Research School NUTRIM, Maastricht University Medical Centre, Maastricht, Netherlands

**Keywords:** overweight and obesity, children and adolescents, physical activity, motivation, body composition, intervention mapping

## Abstract

Overweight and obesity in children and adolescents is seen as a global health challenge and a priority for prevention (1). To solve such a health issue, we need full understanding of the related health behaviors (and underlying beliefs), and understanding of the biological mechanisms that cause or can prevent the issue. However, for overweight and obesity, drawing a full picture of the exact problem (and the subsequent solution) is difficult. In this paper, we describe how we used Intervention Mapping to develop a theory and evidence-based prevention program targeting overweight and obesity and how we investigated the 1-year efficacy of this program on body composition and physical activity of adolescents. A helpful tool, theoretical, and methodological lessons learned are given from our attempt to contribute to solving the obesity problem.

## Introduction

We (and our children) are becoming heavier, overweight and obesity are established risk factors for chronic metabolic and cardiovascular diseases, and something has to be done to make people healthier. In this paper, we describe how we used Intervention Mapping to develop a theory- and evidence-based prevention program targeting overweight and obesity. We (1) developed a prevention program targeting overweight and obesity, (2) performed a literature study and several cross-sectional studies to investigate whether our proposed ideas were correct, and (3) investigated the 1-year efficacy of incorporating strength exercises in gym classes, combined with monthly motivational lessons to engage in physical activities after school, on body composition and physical activity behavior of adolescents. The developed intervention targeted first-year students in preparatory secondary vocational education (11–15 years of age). Teachers were the program implementers. One part of the intervention involved a 30% increase of strength exercises across the physical education (PE) lessons (about 15–20 min per lesson, or 3 h per week). The other part was based on Motivational Interviewing (2) and was facilitated by a trained mentor or PE teacher. Once a month, a 1-h lesson was used to increase motivation to be more physically active. The intervention period was between March 2015 and March 2016. Nine Dutch secondary schools were randomized into an intervention condition (four schools) or a standard curriculum control condition (five schools). Schools were recruited *via* school management and 695 adolescents participated. Measurements were taken before (T0) and directly after (T1) intervention [for a full overview of the intervention, see the open access design paper by Ten Hoor et al. (3)]. We found that strength exercises during PE classes in high schools improve body composition and probably promote physical activity. A helpful tool, we describe the development of our ideas, and theoretical and methodological lessons learned are given from our attempt to contribute to solving the obesity problem.

## The Obesity Problem—Biological and Psychological Perspectives

As being too heavy (or becoming too heavy) is a consequence of an imbalance between energy intake (food intake), and energy expenditure (physical activity, exercise, but also resting metabolic rate, and the cost of ingestion/digestion), the common obesity-prevention adage is to eat less (and healthier) and to exercise more. However, to solve the obesity problem, it has repeatedly been proven that rapid weight loss is a “relatively easy,” but often incomplete and often only short-term solution.

By eating less, preferable short-term changes in body mass adjusted for height (body mass index or BMI; used to determine weight status) can be achieved in younger adults, but several metabolic and hormonal mechanisms often cause a fast weight regain. By quickly losing weight by caloric restriction, one’s energy expenditure also adjusts to a lower energy intake making long-term dieting a necessity for the maintenance of lost weight (4). At the same time, adaptive hormonal responses influence appetite and satiety (5). Another hypothesized cause of a fast weight regain is the screaming fat cell hypothesis: by quickly losing weight, the size but not the number of fat cells reduces. These adipocytes get “stressed,” and try to compensate by an increased uptake of glucose and fatty acids (6).

The other part of “eat less, exercise more,” i.e., being more physically active, might seem easier to achieve, except that it is not. Overweight youngsters can often not compete with normal weight youngsters when it comes to aerobic exercises (7) and are more vulnerable to injuries when engaging in aerobic exercises (8). Additionally, because children or adolescents who are overweight or obese often have less mastery experiences when engaging in aerobic exercises, many psychological theories predict disadvantageous physical activity outcomes for those people. With lower relatedness, competence, or autonomy when it comes to physical activity, Self Determination Theory (9) suggests that the motivation to be physically active is either absent (a-motivation), or extrinsically focused. Extrinsic motivation often results in potential short-term increases in physical activity, but not long-term. Based on negative past-performances, the Reasoned Action Approach (10) predicts lower physical activity attitudes, perceived norms, or self-efficacy, resulting in lower intentions to be physically active, and a decreased physical activity behavior. As a result of inferior physical activity performance compared with others, the Social Comparison Theory (11–13) suggests that this lower performance on a specific dimension (in this case: the physical activity dimension), will cause a shift in focus to other behavioral dimensions where superior performances can foster positive outcome expectations (e.g., being better in guitar playing, or geography). In conclusion, losing weight by eating less and being more physically active might be a short-term solution to being overweight or obese, but is often not the solution to overweight and obesity prevention and treatment, and its related diseases on the long term.

### Biology and Psychology Combined

One could question if the focus in obesity prevention should be on weight or on health as overweight and obesity are seen as a threat to an individual’s health. Impaired physical and psychological health has furthermore been associated with direct [health care (14)] and indirect (productivity or absenteeism) consequences on a societal level (15). All these negative implications might continue during and worsen throughout adulthood (16).

From a health perspective it is not weight but a healthier body composition (i.e., an increase in fat-free mass and/or reduction in fat mass percentage) that needs to be considered (17). Body weight adjusted for height (BMI) is commonly used to determine whether an individual has a healthy or unhealthy weight. Although BMI is a good and simple tool for risk estimates in large populations, it is not the right tool for individual metabolic and mental health evaluations (18) and might have stigmatizing effects on one’s health in later life (19).

Overweight and obesity are seldom caused by the individual only but also by environmental factors (20, 21): from people influencing overweight and obesity at most proximal environmental levels like the parents or peers (22, 23), to societal levels [see, e.g., Borys et al. (24) for an overview of the EPODE approach].

Related to all previous arguments, we advocate that weight loss interventions should become health interventions. Focusing on weight and weight loss might have stigmatizing effects, adversely affecting an individual’s psychological well-being (25, 26). Health care settings, including weight loss interventions are (unintentionally) a significant source of weight stigma. It is often believed that weight stigma triggers weight loss, but there is no evidence to confirm this belief (26). By focusing on health instead of weight, weight stigma might be reduced.

## A New Interdisciplinary Approach

In our program, we assumed that there should be a focus on improving body composition for all youth, rather than only focusing on increasing leanness among those with overweight. Additionally, we suggested extra focus on mastery experiences within the field of physical activity, and optimal social comparison conditions. In other words, the aim was to focus on what people want to do to become healthier (or maintain health) instead of what they have to do.

In the proposed approach, the focus was on strength exercises in high schools to improve body composition in 12- to 15-year-old adolescents. From a physiological perspective, it is known that youngsters who are overweight or obese are generally stronger in absolute terms, i.e., they do not only have a higher fat mass but also a higher fat-free mass (27). Therefore, they are often better in strength-related exercises compared with aerobic exercises and they are often better in strength exercises compared with their normal weight peers, making them—under the right circumstances—more motivated to engage in resistance exercise and ultimately maintain a physically active lifestyle. Additionally, strength exercises can improve body composition. A higher fat-free mass will result in an increase in basal metabolic rate and total energy expenditure [see also Ref. (3, 28)]. In addition, a lower fat mass percentage improves several cardiovascular risk factors.

The intervention was executed in high schools. To reduce stigma, and to optimally use social comparison, the focus was not on weight or adolescents with overweight or obesity, but on health, and on all youngsters. The PE teachers integrated at least 15 min of strength exercises in their PE lessons (three times per week). This proportion was based on the feasibility of integrating strength exercises into the standard curriculum. To motivate students to be more physically active after school, and to improve the determinants of their physical activity behavior, we included a motivational intervention once per month [see also our design paper by Ten Hoor et al. (3); open access]. These motivational lessons were based on motivational interviewing (2) and facilitated by a trained mentor or PE teacher. In the first 5 months, an extra monthly online motivational lesson was given. Together with the feelings of competence and relatedness students experience during the PE lessons, the complete program, therefore, aimed to improve the three basic psychological needs required for autonomous motivation according to Self-Determination Theory (29).

## Main Empirical Findings

This program did not immediately start with the execution of this intervention. In considering strength exercises in health behavior change interventions targeting overweight and obesity, we followed the iterative steps of the Intervention Mapping protocol (21, 30). We systematically mapped what is known about the differential psychological consequences of strength vs. aerobic exercises. In a systematic literature research and meta-analysis (31), we found that strength exercises may have positive effects on a number of psychological outcomes in people who are overweight or obese. These effects, however, seemed often comparable to those of aerobic and diet interventions. The small and heterogeneous evidence base implied an urgent need for more research.

In a related—additional—cross-sectional study (ten Hoor, Plasqui, et al., submitted)^1^, we tested our chain of assumptions. This was in a different population, but provided some relevant data to inform the intervention with adolescents. We confirmed that overweight people have a higher fat-free mass compared with lean people. This was in line with biological insights. Additionally, we have shown that people with a higher fat-free mass are stronger (in absolute sense) compared with those with lower fat-free mass, and are better in strength exercises than they are in aerobic exercises. We have also confirmed that mastery experiences (in this case, resulting from successfully engaging in strength exercises as opposed to aerobic exercises) improve psychological outcomes. Finally, we have shown that overweight people enjoy strength exercises more than normal weight people, mediated by fat-free mass and muscle strength. This series of studies demonstrated the chain of relationships empirically.

Parents have a crucial role in their child’s physical activity-related behavior. We therefore examined parental attitudes about physical activity behavior in general and aerobic and strength exercises in particular (28). Although strength exercises evidently have both physiological and psychological health benefits across all ages, they are erroneously considered to adversely affect health status in youngsters. We found that parents consistently reported a positive attitude toward aerobic exercises, but a less positive, neutral attitude regarding strength exercises. Parents indicated more often that their child was not allowed to participate in strength exercises than in aerobic exercises and considered strength exercises to interfere with optimal physical development. We suggested testing interventions to increase parents’ understanding of the advantages of and possibilities (e.g., facilities) for strength training and the benefits of strength exercises on their child’s health.

In a cluster randomized controlled trial (ten Hoor, Rutten, et al., submitted)^2^, we examined the efficacy of a physical activity program combining strength exercises and motivational aspects in the school setting. We chose this setting to avoid parental influence on the strength exercise component. Within the school setting, and by not only focusing on adolescents with overweight or obesity but on all adolescents, social comparison was optimally used and negative stigma was minimized. With the strength exercise focus, we aimed to improve body composition (by improving fat-free mass), and mastery experiences of adolescents with overweight or obesity. The motivational aspects focused on improvements in physical activity motivations and physical activity behavior in daily life. After 1 year, this study resulted in favorable changes in body composition and physical activity behavior in the intervention group compared with a standard curriculum control group. All adolescents became less physically active after 1 year, which is seen often in youth (32, 33). However, the PA level in the intervention group decreased significantly less as compared with the control group. Our results also indicate that the use of strength training at school alongside a motivational intervention can induce a change in activity levels, also outside PE classes. Several meta-analyses have shown that any improvement in PA behavior may have large beneficial effects (34). Based on our findings in this last study, and earlier performed studies, we concluded that strength exercises might be a valuable contribution to a child’s physical activity possibilities (e.g., facilities) and behavior.

## Theoretical Considerations

In search for an obesity prevention program, many decisions were made. The development of our intervention was based on the Intervention Mapping protocol (21, 30). With this, we described the iterative path from problem identification to problem solving, or mitigation. The six steps of Intervention Mapping comprise several tasks, each of which integrates theory and evidence. The completion of the tasks within a step created a product that guides the subsequent step. The completion of all of the steps served as a blueprint for designing, implementing and evaluating our intervention based on theoretical, empirical and practical information.

### An Interdisciplinary and a Socio-Ecological Approach

Interdisciplinary research, as stated by The Young Academy (35), is often seen as nothing more than a “multidisciplinary” combination of disciplinary perspectives. True interdisciplinarity goes a step beyond this; a crucial step. Interdisciplinary research characteristically involves a change in scholarly identity. This can be defined as “the interplay between the questions that researchers pose, the methods that they use, and the outcome measures that they employ” [(35), p. 7]. The authors state that “a change in scholarly identity can have both beneficial and adverse effects” and they have identified a number of major problems: the disciplinary focus of most funding bodies; the enormous time investment required to familiarize themselves with insights from outside their own discipline; cultural differences between disciplines; and friction with an academic infrastructure that is organized largely into disciplines, especially with regard to educational matters. There are good reasons to remove or at least minimize these obstacles. “Interdisciplinary research makes a major contribution to scientific innovation, leads to greater breadth and depth in individual disciplines, generates cross-disciplinary knowledge, and often plays a vital role in analyzing the major challenges facing society” [(35), p. 7].

In this project, we managed to avoid most of the problems as mentioned above and to profit from the beneficial effects of true interdisciplinarity. The primary investigator was trained as a biologist and psychologist and was able to integrate state of the art knowledge from both disciplines. The coinvestigators contributed from biological, human movement, physiological, child health care, social psychological, and health promotion sciences, without serious frictions. One binding factor was the societal relevance of challenges we faced (from different perspectives) related to obesity in youth.

Next to an interdisciplinary approach, we applied a socio-ecological approach. Socio-ecological models suggest that intervention development should include (or at least consider) all possible stakeholders: not only at the individual level but also at environmental levels (21): interpersonal, organizational, community, and policy levels. Including parents into the program was considered (interpersonal level), but this was not directly necessary. However, future research and interventions are needed (see also Section “Future Research and Implementation”). Although parents are less positive about strength exercises compared with aerobic exercises, and although they play an important role in a child’s physical activity behavior, it was decided to initially work with high schools and PE teachers (organizational level) for three reasons: first, social comparison is part of typical classroom settings and therefore unavoidable in the school setting (36). Second, PE teachers are aware of the benefits of strength training, are able to teach, or emphasize, the right techniques, and are able to provide qualified supervision. The methods used to further prepare the PE teachers for the intervention are facilitation and participatory problem solving [see Bartholomew Eldredge et al. (21), pp. 378 and 391]. The teachers are instructed about the program, participate in workshops to improve their motivational speaking skills, and are provided with materials to make them able to include strength exercises in their lessons. Furthermore, the PE teachers received a book with strength exercises and games as inspirational material. This inspirational material was not only based on literature, but also on ideas from experts in the field, and from the PE teachers themselves Third, when adolescents participate in strength exercises in high school and have positive experiences, they could discuss this with their parents, possibly curving their parents’ attitudes into a more positive direction. For the future, developments at the organizational and policy level will become relevant, such as training of PE teachers as well as activities initiated at the city level to promote exercise behavior in children and adolescents (see Future Research and Implementation).

### Social Comparison Theory

We developed a program for adolescents with overweight or obesity, but involved non-overweight peers for social comparison purposes (interpersonal level; note that the non-overweight peers also benefitted from the program.) We did not suggest promoting “outperforming others,” but promoting “mutual appreciation,” both related to social comparison theory and self-determination theory. Outperforming others might relate to more controlled types of motivation, while a positive comparison with others for youngsters who are used to only experiencing negative comparisons might result in more autonomous motivations. The relation between social comparison and self-determination is an under-investigated area. Some authors [e.g., Ref. (37)] have suggested that stimulating social comparison may have detrimental effects on autonomous motivation. However, O’Keefe et al. (36) suggest that social comparison is part of typical classroom settings and therefore unavoidable. Moreover, Senko et al. (38) argued that normative-based performance goals often facilitate classroom achievement. Standage et al. (39) found that perceptions of competence and relatedness are more predictive of self-determined motivation than autonomy, but also that normative feedback that is repeatedly negative will lead to a-motivation. We think that, next to promoting autonomy [e.g., by giving youngsters choices (29)], positive social experiences of overweight youngsters with resistance exercises may increase their perceptions of competence, their self-worth, and in time, their intrinsic motivation for exercise. In the present intervention, having youngsters compete as teams in multicomponent exercises might have encouraged interpersonal appreciation of various skills (e.g., speed vs. strength). However, the relation between social comparison theory and self-determination theory has rarely been studied empirically (40).

### The Theory of Expanded, Extended and Enhanced Opportunities (TEO)

Recently, Beets et al. (41) argued that the focus of physical activity interventions always should be on (1) expansion of opportunities to be physically active, (2) extension of available opportunities to be physically active, and/or (3) enhancement of the physical activity possibilities and/or opportunities (e.g., facilities). This TEO succeeds in making physical activity behavior more understandable and adds to the bigger picture of understanding obesity and obesity-related behaviors. In our program, we have added the idea of “Enriched Opportunities” of currently available physical activities: we replaced good physical activities by better ones (for people with overweight or obesity). This has been done, prior to expanding, extending, or enhancing the current physical activity opportunities.

## Methodological Considerations

When schematizing our thoughts to solve the obesity problem, there is no straight line between the box “problem” and the box “solution.” Moreover, in each step of the iterative process from “problem” to “solution,” decisions were made, influencing subsequent steps in the process, or sometimes even impacting the entire direction (see our example below where we shift from the obesity clinic to a setting where social comparison played a larger role).

### Study Populations

In our cross-sectional study (ten Hoor et al., resubmitted), linking the biological outcomes with the psychological outcomes, only young adults (18–30 years of age) were included, even though the main focus in the program was on young adolescents aged 12–15 years. This study was in a different population, but provided some relevant data to inform the intervention with adolescents. The measurements during this study were mostly gold standard measurements (VO_2_ max test for aerobic capacity; underwater weighing for body composition; 1-Repetition-Max tests to measure maximal muscle strength), but also considered as being of high intensity, too invasive, difficult or not validated, or even health threatening for youth during growth (42). Less-reliable measuring instruments could have been used in young adolescents (as opposed to young adults). However, as we assumed that the mechanisms would have been the same in youngsters vs. young adults (combined with ethical considerations and easier accessibility), we chose to examine the relationships between the biological and psychological outcomes in young adults, with gold standard outcomes.

Initially, the idea of adding strength exercises to an adolescent’s physical activity was focused on people with overweight or obesity only. Therefore, we first focused on performing our experimental studies in a clinical setting. During the process, and while the program ideas evolved, it was decided to shift our focus from the obesity clinic to a setting where social comparison played a larger role (see also theoretical considerations). Because of this shift, we were not able to perform a classic randomized controlled trial at the individual level, but were forced to cluster in groups of participants. With α = 0.05, power = 0.90, and an assumed small-to-medium effect size (*d* = 0.35), 172 participants per group were needed for a classical RCT. However, in view of the clustering of students within schools and randomized assignment of schools (cluster randomized trial) a sample size of 600 was aimed at to adjust for the design effect arising from clustering (43). The sample size was further increased to 700 to accommodate 15% dropout (although all available data from all participants would be included into the analysis). Although our sample size was quite large, our study was underpowered both due to the larger than expected dropout or missingness and due to the clustered data structure (students nested within schools, randomization of schools; see also measurement issues).

Due to this shift to schools, and our adjusted aims, we did not focus only on adolescents with overweight or obesity but on all adolescents (all adolescents would benefit from the program if effective, including adolescents with a higher BMI). However, in this approach we were able to take baseline BMI into account as a covariate during our analyses to examine differential effects of the intervention due to weight differences. To recruit a population with a slightly higher BMI, we recruited mostly at schools with Lower Vocational Education. These schools are often characterized by adolescents from lower SES households and having a higher BMI. There is a possibility that the baseline characteristics and intervention effects are different when measured in other populations.

### Measurement Issues

The shift from a clinical setting to high schools also caused a shift in the methods that were used for our evaluative measurements. Although gold standard techniques were preferred, these were often too expensive, or difficult to execute in larger populations. It was important to be able to measure both body composition and physical activity behavior (our primary outcomes), but also strength, aerobic capacity, and psychological determinants in large groups of 12- to 15-year-old adolescents in the school setting. All measurements were chosen by considering burden to the participants as well as validity and reliability.

#### Body Composition Considerations

Although there are many techniques to measure body composition [e.g., DXA, hydrostatic weighing, air-displacement plethysmography, deuterium dilution, and multicompartment models, see also (44, 45)], many of those techniques are costly, time-consuming, and logistically challenging to use in large groups of children and adolescents (45, 46). As with all techniques, these also rest on assumptions by which raw data are converted to final values (47) by use of hydration factors [e.g., Ref. (48), or fixed densities of fat- and lean mass (49)].

We used both the skinfold measures and the deuterium dilution technique. Skinfolds are a simple, safe, non-invasive, and widely used technique to measure body composition in larger groups of children and adolescents (46, 47). Originally, skinfold measures were used to estimate body density [using body density regression equations derived from gold standard techniques, see, e.g., Ref. (49, 50)], after which Siri’s (51) equation was used to calculate fat- and fat-free mass. However, equations are population specific, and during maturation, the relation between skinfold thickness and one’s subcutaneous adipose tissue distribution strongly depends on biological age (52–54). Measuring body composition by skinfolds is an indirect method. The measurement might suffer from additional errors while collecting the data. Wells and Fewtrell (47) described that intra- and interobserver error are low compared with between-subject variability, but higher in obese youngsters. The overall error further increases when (a) the collected skinfold values are converted to body density or (in this case) total body water, and (b) these data are converted to fat- and fat-free mass using hydration factors [see, e.g., Ref. (48)], or fixed densities of fat- and lean mass (49).

The more sophisticated method that we used is deuterium dilution (44, 55). We asked students to hand in a baseline urine sample, after which they drank 75 mL of deuterium-enriched water, increasing the deuterium body concentration with 100–150 ppm. At the end of the school day (a minimum of 4.5 h later), a second urine sample was collected. To calculate total body water, the two urine samples (baseline and enriched) are analyzed using isotope ratio mass spectrometry. From total body water, fat-free mass was calculated using age-specific hydration fractions of fat-free mass (48). Compared with underwater weighing (which is still considered a gold standard measurement), deuterium dilution is a reliable method to assess fat mass percentage in normal weight and obese subjects (56), showing the same changes in fat-free mass over time (57). The technique is not often applied in large studies as it requires specific expertise and equipment and is hence relatively expensive. However, this technique was most suitable in our study as it is accurate, non-invasive, requires minimal subject cooperation, and can be applied outside laboratory settings (47).

#### Strength Measurement Considerations

The most frequently used method to measure strength is the handgrip dynamometer because of its cost-effectiveness, simplicity, and portability (58). However, movement patterns performed during the execution of the handgrip test are not comparable to movement patterns of larger muscle groups, or performed in daily life or exercise training programs (59). To partly overcome this limitation, we evaluated the back–leg–chest (BLC) dynamometer as an effective, simple, and portable way to test total body strength [a simplified explanation: we asked students to stand on the base of the BLC dynamometer with flexed knees (ca. 30^○^) and asked them to lift a handle that was connected to the base with a continuous vertical motion by extending the knees, hips, and lower back—for a full explanation, see Ref. (60)]. The apparatus induced execution of static contractions, which are required less often in daily life compared with dynamic contractions. The BLC dynamometer provided reasonably reliable test–retest measurements of BLC strength in healthy adolescents and adults and was therefore considered as useful additional tool in our study (3).

#### Physical Activity in Daily Life Considerations

For physical activity in daily life, the Actigraph GT3X accelerometer (Actigraph, Pensacola, FL, USA) was used. The Actigraph GT3x triaxial accelerometer is a small device and measures acceleration in three directions (vertical, antero-posterior, and mediolateral). The Actigraph was chosen as it is the most validated triaxial accelerometer that is currently commercially available [see, for many references, Plasqui and Westerterp (61)]. Also, activity patterns can be determined as child-specific cut-off points for low-, moderate-, and high-intensity physical activity are available. Although this was a relatively simple measurement with a low burden to participants, compliance was lower than expected: some students forgot to wear the accelerometer (especially during weekend days), and some complained that they did not like to wear the elastic band with the device because it did not match their choice of clothing, it hurt their back or was irritating in another way.

#### Questionnaire Considerations

To keep the length of the included questionnaire relatively short, it was chosen to include questions relating to the Reasoned Action Approach, Self Determination Theory, and Social Comparison. Although it would have been interesting to include other clinical outcome measures (e.g., quality of life, self-esteem, mood, stress), this would have increased the length of the questionnaire substantially, and with that the burden on the participants.

#### Aerobic Capacity Considerations

To measure aerobic capacity, the shuttle-run test was used. For this test, students ran back and forth over a distance of 18 m (officially the distance for a shuttle-run test is 20 m, but because not all schools have a 20-m gym court, this test was taken over 18 m—therefore, comparisons within this study are valid, but the results cannot be compared with other studies). The running speed was determined by the interval between two sound signals (“beeps”). Every minute, the speed increased by shortening the interval between two beeps. When a student fails to reach the 18-m line at the sound signal two times in a row, the test stops for this individual. The shuttle-run test has a moderate-to-high validity for estimating maximum aerobic capacity and can easily be executed in larger groups (62). Submaximal and maximal tests using treadmill, or cycle ergometer, or tests where heart rate monitors were required were seen as unreachable in classroom setting. Compared with the Cooper test (where students are asked to run for 12 min and where the distance was used as proxy for aerobic capacity), the shuttle-run test was less likely to be influenced by weather conditions.

### Missingness Because of the Intensive Measurements

Although both of our main outcomes (body composition and physical activity behavior) measuring techniques (deuterium dilution and accelerometry) are accurate, and acceptable in all age groups, the method is relatively expensive and thus not often applied in larger studies. A limitation was that, due to the nature of these measurements (i.e., two small urine samples are required), many students decided to not participate in this measurement (either at T0, T1, or both), causing missingness. While all available data were included into the analysis using a method that is valid under so-called missingness at random (MAR, missingness depends on observed variables such as age or pretest if posttest is missing), we cannot rule out bias arising from missingness not at random (MNAR, missingness depends on unobserved variables such as posttest if posttest is missing). Unfortunately, MNAR cannot be detected or adjusted for. At best, complex methods can be used to explore the robustness of results against various patterns of MNAR missingness (63).

## Future Research and Implementation

We have processed evaluation data on secondary physiological and psychological outcomes, and are collecting data on teachers’ experiences with our program. In addition, and for a long-term effective strength-based physical activity program, there are still several questions that need to be answered.

### Future Research

#### Psychological Behavioral Determinants

From our systematic review and meta-analysis, we concluded that strength exercises are a viable alternative to or addition to diet and/or aerobic interventions when it comes to improving psychological outcomes, but more research is necessary. In the literature, studies reported only on a limited range of psychological outcomes. The reported psychological outcomes were mostly clinical outcomes or markers of quality of life. Measuring self-determination concepts as psychological constructs might give additional information about the effects of exercise training to be considered alongside that obtained from current clinical and quality of life measures.

#### Feedback and Body Composition

We also suggested giving feedback on body composition instead of weight loss to improve psychological outcomes. Pescud et al. (64) reported that feedback on body composition is useful as a “surrogate” for feedback on weight loss, which motivated participants to continue participating in strength training exercises. Gaining strength, and ultimately obtaining a healthier body composition, might lead to a higher resting metabolic rate, increased total energy expenditure, and a decreased chronic diseases risk (17). Thus, when participants in a strength training program become stronger, this could also lead to (long term) positive changes in body composition and health. However, these positive effects are often not reflected in reported short-term psychological outcomes of strength training as compared with other interventions. Related to this, two more recommendations for future research arise. First, as described under theoretical considerations, the relation between social comparison theory and self-determination theory has rarely been studied empirically (40) and needs to be further investigated. Second, valid and reliable methods to measure body composition should become more accessible to the public.

#### Parental Attitudes and Influence

In the study examining parental attitudes regarding strength training, we concluded that future interventions should increase parents’ understanding of the advantages and possibilities (e.g., facilities) of strength training on their child’s health and that strength training can be fun. Without parental support, it will be more difficult for (overweight) youngsters to engage in resistance exercises (65).

#### Tailoring

Further research is required on how possible program characteristics (e.g., intensities, quantities, form of exercise, feedback mechanisms) can be tailored to the individual (e.g., for the same exercise, an overweight adolescent lifts a heavier weight than a lean adolescent) or group level [e.g., girls may prefer different resistance exercises than boys (66)].

#### Replication

Lastly, future randomized controlled trials should replicate our findings and evaluate the immediate and long-term effectiveness of our approach. This can be done in high schools, but also in other settings and age groups (e.g., primary schools, clinical setting).

### Implementation

Once an intervention has been created, a solid diffusion and implementation process is vital to ensure program success (21). Without implementation, the intervention will not have any chance of impact on determinants, behaviors, or health. The basic idea of our approach is simple and easily implementable. For future implementation infrastructures at schools (including the playgrounds) can be optimized, not only in high schools, but also in primary schools.

For the future, developments at the organizational and policy level will become relevant, such as training of PE teachers as well as activities initiated at the city level to promote exercise behavior in children and adolescents. PE teachers can be informed and educated about the background of the strength exercises, with guidelines and suggestions for practice. A work book with exercises is freely available but it was noticed that teachers themselves can easily come up with new ideas about strength exercises in the lessons the moment they understand the principle and find out that the students react positively, especially the students with overweight [see the additional file of our open access design paper by Ten Hoor et al. (3)].

Outside the school setting, different sports in which pure physical strength and/or body mass are beneficial (e.g., rugby, judo) could be systematically promoted as an alternative for youngsters with overweight. Fitness centers provide strength training possibilities but they are often not accessible for youngsters. In the future, schools may collaborate with fitness centers to create optimal circumstances, or schools themselves may provide fitness equipment for their students.

## Conclusion

We developed, implemented, and evaluated an interdisciplinary theory- and evidence-based program that positively influenced body composition and physical activity. This might not be a direct solution to combat obesity, but it might help in the long term with the prevention of obesity-related health issues. We suggest adding strength exercises to children’s and youngsters’ physical activity possibilities: as long as strength exercises are performed under qualified supervision, they might have positive long-term health benefits.

## Author Contributions

GH, GP, AS, and GK conceived of, designed, and coordinated the study. GH drafted the manuscript. All authors read and approved the final manuscript.

## Conflict of Interest Statement

The authors declare that the research was conducted in the absence of any commercial or financial relationships that could be construed as a potential conflict of interest.

## Footnotes

^1^Ten Hoor GA, Plasqui G, Schols AMWJ, Kok G. A benefit of being heavier is being strong.

^2^Ten Hoor GA, Rutten GM, Van Breukelen GJP, Kok G, Ruiter RAC, Meijer K, et al. Strength exercises during physical education classes in secondary schools improve body composition: a cluster randomized controlled trial.
